# Efficacy of topical vancomycin in preventing surgical site infection following fracture surgery: a systematic review and meta-analysis

**DOI:** 10.1302/2633-1462.710.BJO-2026-0126.R1

**Published:** 2026-10-02

**Authors:** Neel Badhe, Amelia Hutton, Abbas See, Benjamin J. Ollivere, Ben Arthur Marson

**Affiliations:** 1 Department of Orthopaedics and Trauma, Academic Unit of Injury, Recovery and Inflammation Sciences, University of Nottingham, Nottingham, UK; 2 School of Medicine, University of Cambridge, Cambridge, UK; 3 School of Medicine, University of Nottingham, Nottingham, UK; 4 Musculoskeletal, Surgery, Inflammation and Recovery Theme, NIHR Biomedical Research Centre, University of Nottingham, Nottingham, UK

**Keywords:** Vancomycin, Fracture-related infection, Surgical site infection, fracture surgery, vancomycin powder, surgical site infection (SSI), Fracture-related infection (FRI), randomized controlled trials, lower limb fractures, infections, nephrotoxicity, organisms, nonunion

## Abstract

**Aims:**

Fracture-related infection (FRI) is a major cause of morbidity following limb fracture surgery. Topical vancomycin has gained popularity as an adjunct to systemic prophylaxis; however, concerns regarding nephrotoxicity, impaired bone healing, and wound complications remain. This review aims to evaluate the efficacy and safety of intraoperative topical vancomycin powder in preventing FRI in patients undergoing surgical management of upper or lower limb fractures.

**Methods:**

A systematic review of randomized controlled trials (RCTs) was conducted in February 2026. Comprehensive searches of Medline, EMBASE, and Cochrane CENTRAL were performed. Trials were included if they randomized patients with limb fractures between intraoperative topical vancomycin powder and no vancomycin, placebo, or standard care. The primary outcome was FRI or deep surgical site infection (SSI). Secondary outcomes included time to FRI, organism profile, antimicrobial resistance, superficial SSI, nonunion, revision surgery, wound healing problems, renal impairment, and other reported complications.

**Results:**

Five studies involving 1,373 patients were included. Topical vancomycin significantly reduced FRI rates compared with controls (risk ratio (RR) 0.64 (95% CI 0.43 to 0.96); p = 0.030). A significant reduction in FRIs caused by gram-positive organisms was identified in patients given topical vancomycin (RR 0.41 (95% CI 0.24 to 0.71); p = 0.001). No differences in FRIs caused by gram-negative or resistant organisms were identified. There were no significant differences in superficial SSI, nonunion rates, wound healing, or other complications. Topical vancomycin was not associated with a significant risk of nephrotoxicity.

**Conclusion:**

Topical vancomycin powder may be a safe and effective adjunct to standard systemic prophylaxis for preventing FRI, particularly in reducing gram-positive infections. However, current evidence is limited by a lack of high-quality RCTs and small sample sizes. A large, definitive RCT is needed to determine the efficacy, safety, and cost-effectiveness of topical vancomycin in limb fracture surgery.

Cite this article: *Bone Jt Open* 2026;7(10):1253–1260.

## Introduction

Fracture-related infection (FRI) is a serious postoperative complication and significant cause of morbidity following fracture management surgery.^1^ It occurs in approximately 1% to 2% of closed fractures and in over 30% of open fractures.^2^ FRI is associated with prolonged hospitalization, increased need for further surgery, and substantially higher healthcare costs, with some studies suggesting a two-to-six-fold increase in treatment expenditure.^3-6^ Furthermore, the incidence of FRI has risen by approximately 20% over the last decade and is predicted to continue rising, highlighting the urgent need for more effective preventative strategies.^1,3^

Current prevention protocols involve systemic intravenous (IV) antibiotic prophylaxis alone. However, rising FRI rates, coupled with concerns regarding suboptimal antibiotic penetration into poorly perfused tissues and the risks associated with systemic exposure, have led to the suggestion of more direct antibiotic delivery methods.^7,8^

Routine use of intraoperative local antibiotic therapy as an adjunct to IV antibiotics has been proposed.^9,10^ Local application enables delivery of significantly higher antibiotic concentrations while minimizing systemic concentrations and adverse effects.^11,12^ Several forms of topical antibiotic therapy, e.g. antibiotic-containing beads, and hydrogels, have been investigated,^12-14^ with early studies indicating that aminoglycosides and glycopeptides may be the most promising.^13,15^ In particular, topical vancomycin powder has gained popularity with studies demonstrating reductions in FRIs.^16-18^ However, high-quality randomized controlled trial (RCT) evidence to support its routine use is limited. Additionally, there are concerns regarding impaired bone and wound healing, nephrotoxicity, and the development of antimicrobial resistance.^19-23^

The aim of this systematic review was to identify and evaluate the current RCT evidence for the efficacy and safety of intraoperative topical vancomycin powder for the prevention of FRI following upper or lower limb fracture surgery, compared to no vancomycin, placebo or standard care.

## Methods

A systematic review was conducted on 6 February 2026 to identify RCTs comparing the incidence of FRI with intraoperative vancomycin powder versus placebo, no intervention, or standard care in fracture management surgery. The study protocol was developed in accordance with Cochrane collaboration principles,^24^ was prospectively registered on PROSPERO (CRD420261303128). This review was conducted according to Preferred Reporting Items for Systematic Reviews and Meta-analyses (PRISMA) guidelines.^25^

Comprehensive searches of OVID Medline, OVID EMBASE, and Cochrane CENTRAL databases were conducted using a search strategy with the following key concepts: upper or lower limb fractures; vancomycin; FRI; and RCTs excluding non-human subjects. The complete search strategy for each database is provided in Supplementary Item 1. Additional screening of the grey literature was performed by reviewing conference proceedings from the American Academy of Orthopaedic Surgeons (AAOS), British Orthopaedic Association (BOA), and Orthopaedic Trauma Association (OTA), as well as Google Scholar.

Studies were included if they met the following eligibility criteria: 1) patients with upper or lower limb fractures; 2) randomized patients between intraoperative vancomycin powder and placebo, no intervention, or standard care groups; 3) reported the primary outcome of FRI. Non-randomized studies, lab studies, review articles, and studies not published in the English language were excluded.

Following removal of duplicates, titles and abstracts were independently screened by two reviewers (NB, AH). Full-text articles were subsequently assessed for inclusion by the same two reviewers with discrepancies resolved through discussion.

Data were extracted independently by two reviewers (NB, AH) into a pre-designed extraction table, with disagreements resolved through review by the senior author (BAM). Study characteristics, including patient demographics, injury characteristics, and intervention group size, were extracted from each included study. The primary outcome, FRI/deep surgical site infection (SSI), was also extracted. Extracted secondary outcomes included: time to FRI, species responsible for FRIs, antimicrobial resistance, superficial SSI, nonunion, revision surgery, wound healing problems, renal impairment, and other complications.

Risk of bias was assessed using the Cochrane Collaboration RoB 2.0 tool.^26^ Two reviewers (NB, AH) independently evaluated the risk of bias for each study, and disagreements between individual assessments were resolved through discussion.

### Statistical analysis

Data from comparable RCTs were pooled in a meta-analysis. Heterogeneity was assessed using the I^2^ statistic. We considered I² > 75% as considerable heterogeneity, particularly if it could not be explained by the diversity of the methodological or clinical features of the studies.^27^ The pooled data were analyzed using RStudio v. 4.5.2 (Posit, USA), using fixed- or random-effects meta-analysis models based on heterogeneity. Mean differences (MDs) and risk ratios (RRs) were used for continuous and categorical variables, respectively. Significance was set at p < 0.05. Southampton Wound Scores were converted into a binary outcome, with scores of III to V (discharge, pus, breakdown, or infection) classified as adverse events.^28^

## Results

The PRISMA flow diagram is shown in Figure 1. The initial search of databases and Google Scholar identified 478 records, with 317 remaining after duplicates were removed. Title and abstract screening identified ten potentially relevant records, of which eight records of five studies met the eligibility criteria (Table I). A summary of the study characteristics and reasons for exclusion of articles is provided in Supplementary Item 2. No additional studies were included following review of conference proceedings.

**Fig. 1:**
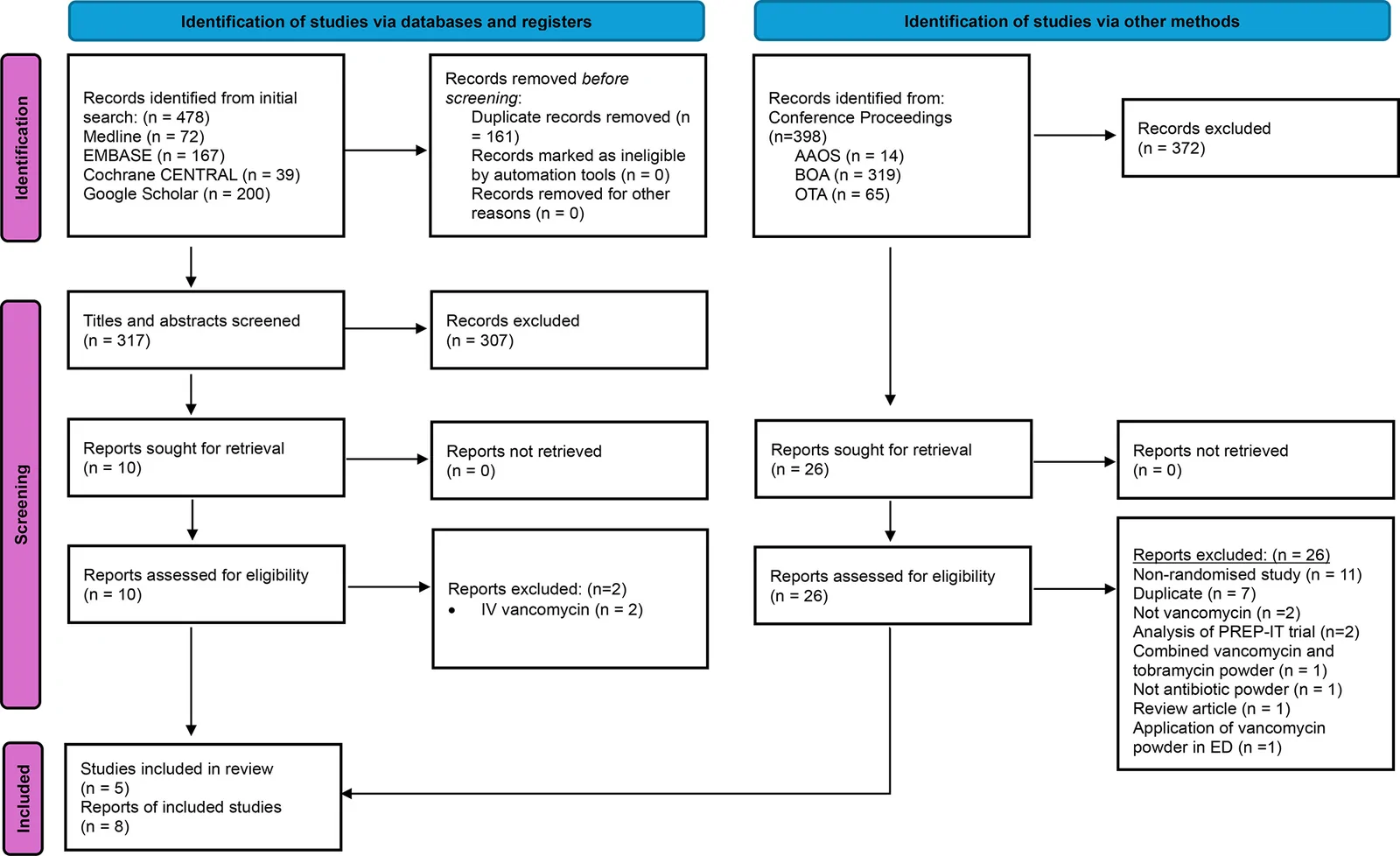
PRISMA Flow Diagram. AAOS; BOA, British Orthopaedic Association; ED, emergency department; IV, intravenous; OTA, Orthopaedic Trauma Association.

**Table I.** The study characteristics, demographics, and relevant outcomes reported in the included studies.

Study	Country	Loss to follow-up, n (%)	Fractures included	Intervention (n)	Control (n)	Universal prophylaxis	Mean age, yrs (SD)	Female, %	Relevant outcomes reported
Acosta-Olivo et al^29^	Mexico	3 (2.3)	Closed ankle fractures	1 g topical vancomycin powder (65)	No topical antibiotics (64)	IV cephalothin 1 g	38.9 (13.8)	51.1	FRI rate, species responsible
Gandhi et al^30^	India	8 (8.3)	Isolated closed limb fractures > 2 wks from injury	2 g topical vancomycin powder (30)	No topical antibiotics (30)	IV ceftriaxone 1 g	40.6 (14.8)	26.1	FRI rate, nonunion, wound healing, other complications
Mohammadhoseini et al^31^	Iran	0 (0.0)	Femoral neck fractures undergoing hemiarthroplasty	1 g intra-articular vancomycin (24)	No topical antibiotics (24)	IV cefazolin 2 g	73.0 (4.5)	58.3	FRI rate, superficial SSI, wound healing
O’Toole et al^8,21,32,33^	USA	56 (5.4)	Tibial plateau or Pilon fracture deemed to be at high risk of infection	1 g intrawound vancomycin powder (481)	No topical antibiotics (499)	Standard infection prevention protocol	45.8 (13.7)	37.0	FRI rate, species responsible, antimicrobial resistance, superficial SSI, nonunion, wound healing, renal impairment, other complications
Rathore et al^34^	India	0 (0.0)	Gustilo-Anderson type I to IIIB open long bone fractures	1 g vancomycin powder at the surgical site (64)	0.9% saline at surgical site (64)	Not stated	43.6 (12.9)	25.0	FRI rate, nonunion, wound healing

FRI, fracture-related infection; IV, intravenous; SSI, surgical site infection.

A total of 1,373 patients were included in this review. Included patients had a mean age of 45.6 years (SD 14.6), and 512 (37.3%) were female. Across the five included studies, three evaluated closed fractures, one focused on open fractures, and one study included patients with ‘high-risk’ fractures (either open or closed). Four studies involved internal fixation procedures, whereas one investigated FRI after hemiarthroplasty. Four studies compared use of 1 g^8,29,31^ or 2 g^30^ intraoperative vancomycin powder with standard IV antibiotic prophylaxis to standard IV prophylaxis alone. Rathore et al^34^ randomized patients between 1,000 mg vancomycin powder and placebo (0.9% saline at the surgical site) for open fractures, but did not report the use of any additional antibiotic prophylaxis.

One study was found to be at low risk of bias.^8^ All other included trials were judged to have some concerns or a high risk of overall bias.^29-31,34^ A summary of the risk of bias assessments of included studies is provided in Supplementary Item 3.

### Outcomes

The number of patients developing FRI or deep SSI during the postoperative period were pooled from all five trials (Figure 2).^8,29-31,34^ There was no significant difference in FRI rates between patients receiving intraoperative vancomycin controls in the closed fracture fixation (RR 0.63 (95% CI 0.36 to 1.08); p = 0.095), arthroplasty (RR 0.50 (95% CI 0.05 to 5.15); p = 0.560), or open fracture groups (RR 0.67 (95% CI 0.37 to 1.23); p = 0.199). However, a significant reduction in the FRI rate was identified in patients receiving intrawound vancomycin powder compared to controls after pooling (RR 0.64 (95% CI 0.43 to 0.96); p = 0.030, fixed effects model).

**Fig. 2:**
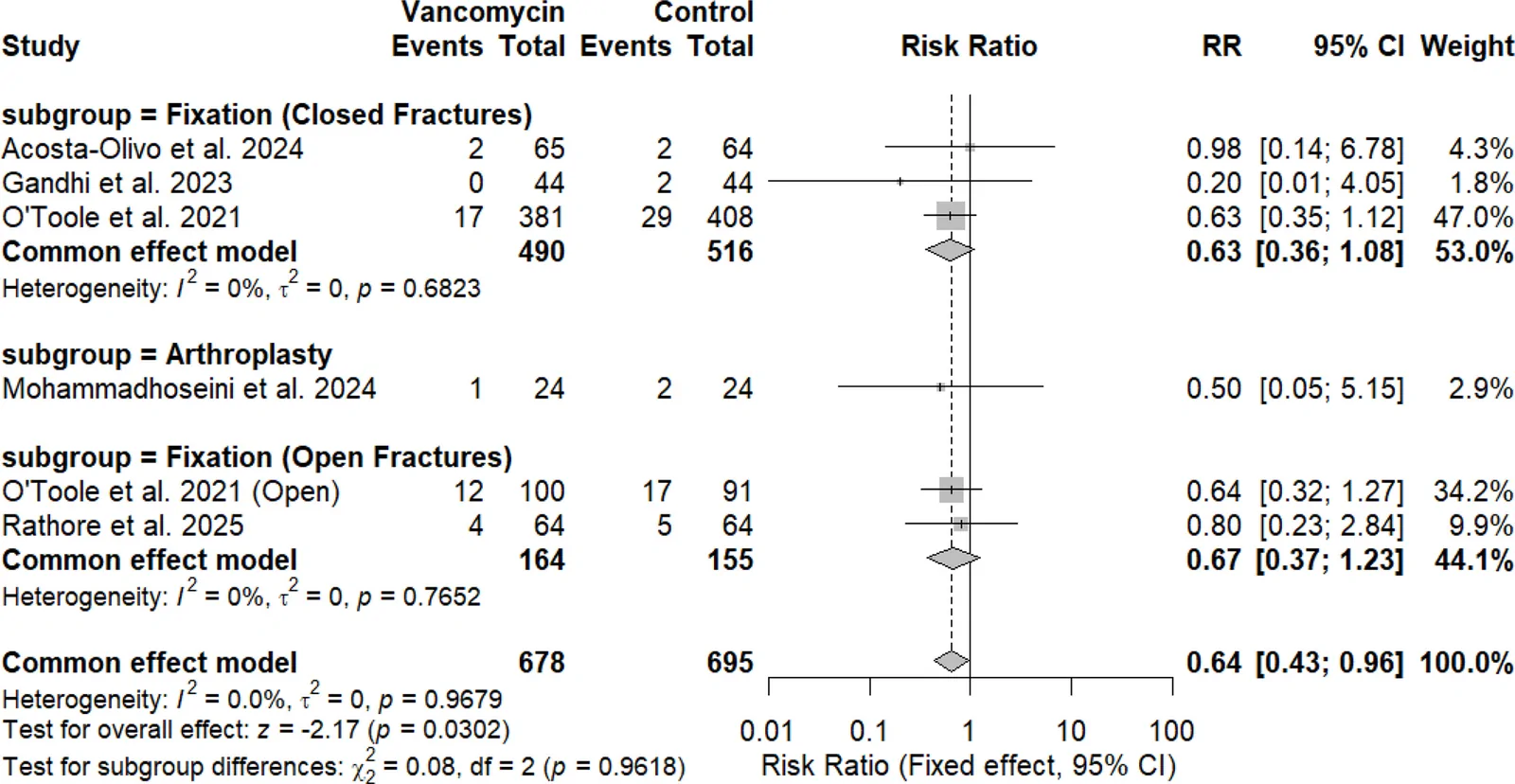
Forest plot showing the risk of developing fracture-related infection in vancomycin compared with control groups. RR, risk ratio.

The number of FRIs that were caused by gram-positive compared with gram-negative organisms were reported in Acosta-Olivo et al^29^ and O’Toole et al*.*^8^ Pooled analysis identified that intraoperative vancomycin powder significantly reduced the number of FRIs driven by gram-positive organisms (RR 0.41 (95% CI 0.24 to 0.71); p = 0.001, fixed effects model), but no difference in the risk of developing gram-negative FRI (RR 1.23 (95% CI 0.55 to 2.77); p = 0.616, fixed effects model) (Figure 3).

**Fig. 3:**
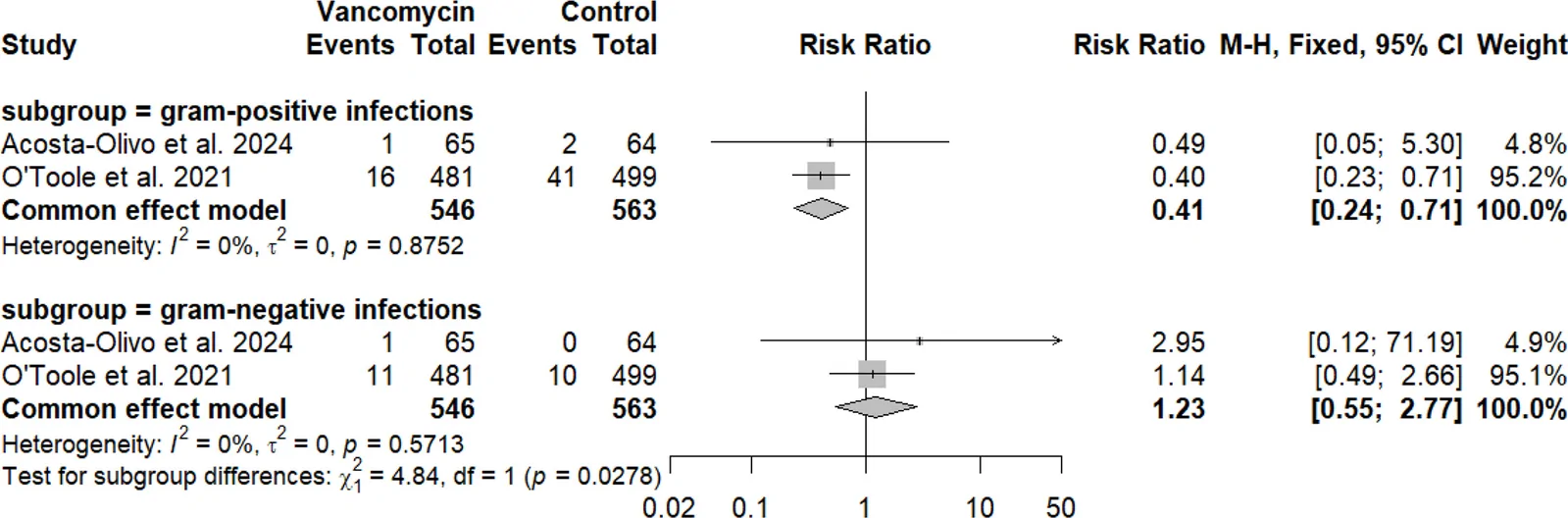
Forest plot showing the risk of developing fracture-related infection caused by gram-positive and gram-negative organisms in vancomycin compared with control groups. M-H, Mantel-Haenszel; RR, risk ratio.

Antibiotic resistance was reported in a secondary analysis of the VANCO trial.^32^ Vancomycin powder had no significant effect on the risk of developing methicillin-resistant *Staphylococcus aureus* (MRSA) (0.9% vs 0.9%; p > 0.999 or vancomycin-resistant *Enterococcus* (VRE) (0.2% vs 0.2%; p > 0.999) but significantly reduced the number of methicillin-susceptible *S. aureus* (MSSA) infections compared with controls (1.4% vs 4.8%; p = 0.01).

The number of patients who developed superficial SSI were pooled from two studies (Figure 4),^8,31^ showing no significant difference (RR 1.28 (95% CI 0.66 to 2.48); p = 0.470, fixed effects model).

**Fig. 4:**
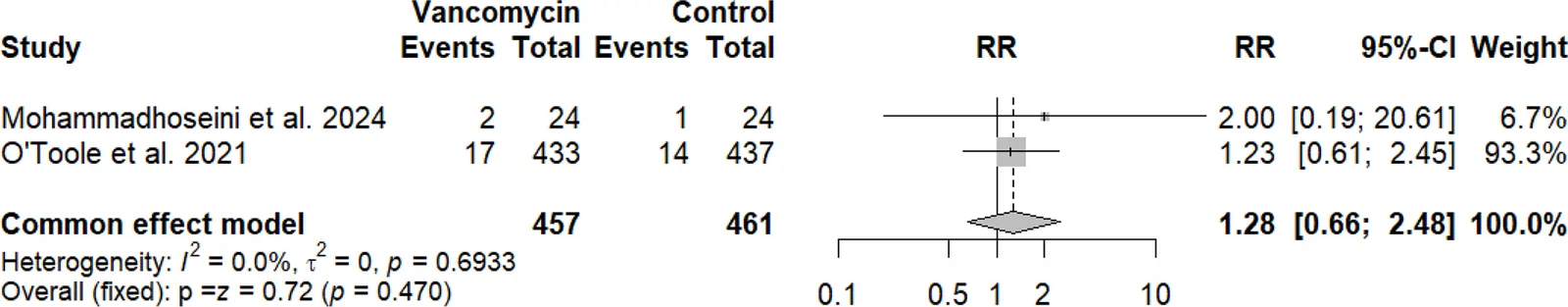
Forest plot showing the risk of developing superficial superficial site infection in vancomycin compared with control groups. RR, risk ratio.

Union status was pooled from three trials (Figure 5),^8,30,34^ showing no significant difference in the proportion of patients experiencing nonunion between vancomycin and control groups (RR 1.37 (95% CI 0.90 to 2.10); p = 0.143, fixed effects model).

**Fig. 5:**
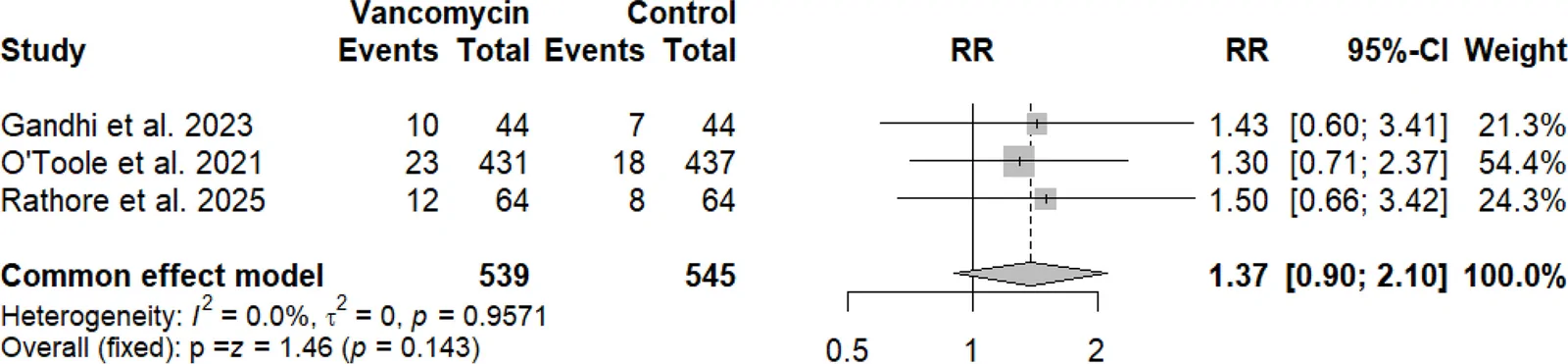
Forest plot showing the risk of developing nonunion in vancomycin compared with control groups. RR, risk ratio.

The number of patients experiencing wound healing-related adverse events was reported in three studies (Figure 6).^8,30,34^ There was no significant difference between vancomycin and control groups (RR 1.00 (95% CI 0.76 to 1.33); p = 0.974, fixed effects model).

**Fig. 6:**
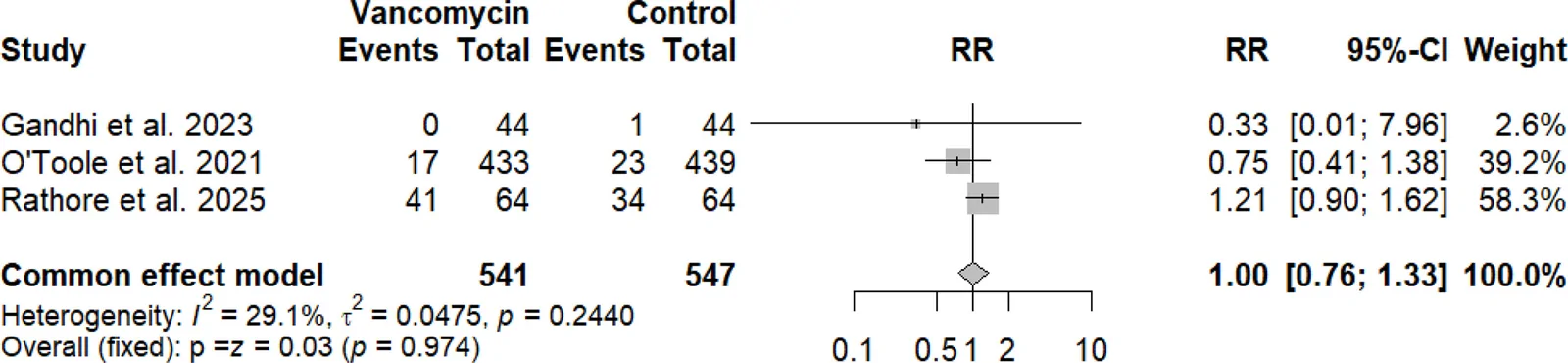
Forest plot showing the risk of developing wound healing-related adverse events in vancomycin compared with control groups. RR, risk ratio.

Renal function, assessed by serum creatinine, was reported in a secondary analysis of 56 patients in the VANCO trial.^21^ All 53 patients with normal baseline creatinine remained normal on postoperative day 1, and at two weeks among 43 with follow-up. The three patients with elevated baseline levels remained elevated. Based on the RIFLE criteria, changes were insignificant in two patients, while one was classified as at risk for renal dysfunction.^35^

Time to FRI or need for revision surgery were not reported in any of the included studies. Additional complications were reported by Gandhi et al^30^ and O’Toole et al.^8^ No significant differences were found in pooled rates of bone lysis, implant loosening, fixation failure, amputation, or death (see Supplementary Item 4).

## Discussion

This systematic review has identified a paucity of high-quality randomized evidence supporting the use of topical vancomycin for the prevention of FRI in fracture management surgery. There was considerable clinical heterogeneity across studies, with significant differences in fracture profile. Additionally, this review only identified one RCT with a low overall risk of bias, with other trials presenting methodological limitations. Given this level of uncertainty, the findings of this review should be interpreted cautiously.

Encouragingly, the use of topical vancomycin does seem to reduce the rate of overall FRI. While no significant difference was identified in any one individual study or subgroup, the overall pooled analysis demonstrated a significant reduction in FRI rates in patients who were randomized to topical vancomycin treatment (p = 0.030). Topical vancomycin seems to be effective in reducing the rate of gram-positive infections (p = 0.001), particularly MSSA.

Our findings are consistent with vancomycin’s mechanism of action. Vancomycin exerts bactericidal effects through disruption of lipid II, a key component of the bacterial cell wall for both gram-positive and gram-negative organisms.^36^ By disrupting cell wall synthesis, vancomycin exposes the cytoplasmic membrane of gram-positive bacteria, resulting in cell lysis.^37^ However, vancomycin’s large molecular size makes it impermeable to the outer membrane of gram-negative species, rendering them ineffective and limiting its effectivity to gram-positive species.^35^

One proposed explanation for the superior efficacy of combined topical vancomycin powder with systemic IV prophylaxis, compared to IV antibiotic prophylaxis alone, is targeted delivery.^38^ Topical vancomycin enables high local concentrations to be achieved at the surgical site and implant interface, which may help to reduce biofilm formation.^39,40^ This is particularly important, given its limited efficacy against established *S. aureus* biofilms.^41^

Only one previous systematic review, conducted by Caruana and Giordmaina^42^ in 2025, has investigated the efficacy of topical antibiotics in preventing FRI in patients with limb fractures. However, this review was limited to open fractures and identified only one trial for inclusion. In contrast, our review evaluated topical vancomycin powder across all limb fractures and identified four additional trials, including Rathore et al,^34^ which investigated its use in open fractures. Additionally, the Caruana and Giordmaina review included cohort studies, introducing the risk of selection bias into their conclusions. Consequently, the results of that review are not comparative to ours. Our review constitutes the highest quality synthesis of all available evidence investigating the use of topical vancomycin powder for preventing FRI in all limb fractures to date. That said, Caruana and Giordmaina^42^ reported similar findings, with reduced SSI rates following topical vancomycin powder treatment in open fractures.

The efficacy of supplemental topical antibiotic therapy for the prevention of FRI in open limb fractures is well supported. Morgenstern et al^43^ reported an 11.9% reduction in infection rates in patients given additional local antibiotics (odds ratio (OR) 0.30 (95% CI 0.22 to 0.40); p < 0.001). However, its routine use for preventing FRI in closed fracture fixation and fracture-related arthroplasty remains underexplored, and warrants further investigation.

There was no significant difference in nonunion rates, however there was a non-significant trend in favour of control groups in all three studies investigating this outcome.^8,30,34^ In vitro studies have shown that vancomycin was associated with dose-dependent death of mesenchymal stem cells,^20^ as well as disruption of osteoblasts function and survival.^44^ Conversely, Rathbone et al^45^ showed that vancomycin was one of the least cytotoxic, and did not significantly affect cell number until very high concentrations. Given the in vitro nature of these studies, it is difficult to draw a clinical conclusion, and highlights the need for further investigation in a larger definitive trial.

This review identified no clinically meaningful nephrotoxicity associated with topical vancomycin powder. Although this outcome was assessed only in a secondary subgroup analysis of the VANCO study, the authors suggest this may reflect minimal systemic absorption, as no patients had detectable serum vancomycin levels (> 5 µg/mL) at one or six to eight hours postoperatively, except for two who also received IV vancomycin.^21^ Additionally, a secondary analysis of the PREP-IT trial found no association between the cumulative dose of intrawound vancomycin powder and drug-induced acute kidney injury in fracture patients (OR 1.08).^40^ These findings indicate that targeted vancomycin powder results in limited systemic absorption and a low risk of nephrotoxicity.

This review is limited by the quality of randomized evidence that is available on this subject. The small number of studies included in the review limits the strength of the conclusions. Additionally, the heterogeneity in fracture types and antibiotic prophylaxis regimens limits the generalizability of our findings. However, this review has been conducted to highest methodological standards, strictly adhering to PRISMA guidelines and Cochrane Collaboration methodological principles, providing the highest-quality synthesis of the available level I evidence.

Topical vancomycin powder may be a safe adjunct to standard systemic prophylaxis for the prevention of FRI in limb fracture surgery. The current evidence indicates that topical vancomycin has a low nephrotoxicity risk, and appears not to affect union or wound healing, or increase other complications. However, high-quality randomized evidence is limited.

In conclusion, there is a need for a large, definitive, RCT to evaluate the efficacy, safety and cost-effectiveness of topical vancomycin for the prevention of FRI in limb fractures. Initial findings are encouraging; however, more robust level I evidence is required to support its routine use for fracture management surgery.

**Take home message**

- Topical vancomycin powder appears to be a safe and effective adjunct to systemic prophylaxis for preventing fracture-related infection in limb fracture surgery, reducing gram-positive infections without increasing the risk of nonunion, wound healing complications, or nephrotoxicity.

- However, high-quality randomized evidence remains limited, and a definitive randomized controlled trial is essential.

## Data Availability

The data that support the findings for this study are available to other researchers from the corresponding author upon reasonable request.
